# Multiscale Agent-Based and Hybrid Modeling of the Tumor Immune Microenvironment

**DOI:** 10.3390/pr7010037

**Published:** 2019-01-13

**Authors:** Kerri-Ann Norton, Chang Gong, Samira Jamalian, Aleksander S. Popel

**Affiliations:** 1 Department of Biomedical Engineering, School of Medicine, Johns Hopkins University, Baltimore, MD 21205, USA; 2 Computer Science Program, Department of Science, Mathematics, and Computing, Bard College, Annandale-on-Hudson, NY 12504, USA; 3 Department of Oncology and the Sidney Kimmel Comprehensive Cancer Center, School of Medicine, Johns Hopkins University, Baltimore, MD 21205, USA

**Keywords:** multiscale systems biology, computational biology, quantitative systems pharmacology (QSP), immuno-oncology, immunotherapy, immune checkpoint inhibitor, mathematical modeling

## Abstract

Multiscale systems biology and systems pharmacology are powerful methodologies that are playing increasingly important roles in understanding the fundamental mechanisms of biological phenomena and in clinical applications. In this review, we summarize the state of the art in the applications of agent-based models (ABM) and hybrid modeling to the tumor immune microenvironment and cancer immune response, including immunotherapy. Heterogeneity is a hallmark of cancer; tumor heterogeneity at the molecular, cellular, and tissue scales is a major determinant of metastasis, drug resistance, and low response rate to molecular targeted therapies and immunotherapies. Agent-based modeling is an effective methodology to obtain and understand quantitative characteristics of these processes and to propose clinical solutions aimed at overcoming the current obstacles in cancer treatment. We review models focusing on intra-tumor heterogeneity, particularly on interactions between cancer cells and stromal cells, including immune cells, the role of tumor-associated vasculature in the immune response, immune-related tumor mechanobiology, and cancer immunotherapy. We discuss the role of digital pathology in parameterizing and validating spatial computational models and potential applications to therapeutics.

## 1. Introduction

**Figure 1.**
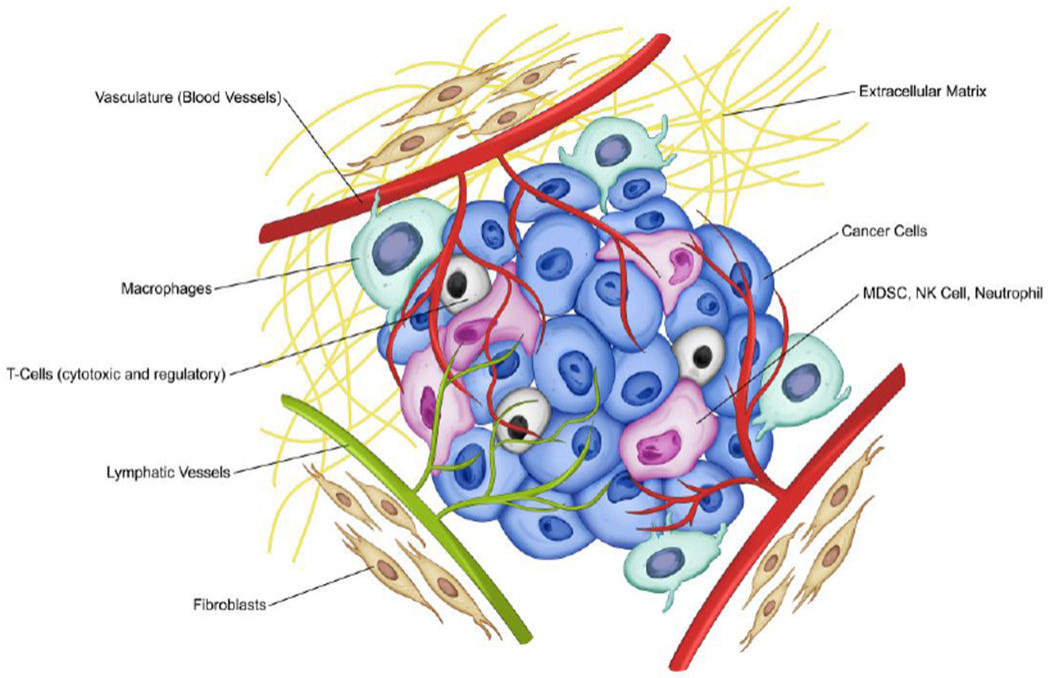
The Tumor Microenvironment (TME). The tumor microenvironment consists of different types of cells (cancer and stromal including immune cells), the extracellular matrix (ECM), and the myriad molecules such as chemokines, cytokines, microRNAs, and growth factors. Cancer cells (including cancer stem cells and progenitor cells), the tumor vessels (blood vessels and lymphatic vessels), immune cells (including tumor-associated macrophages (TAM) and T-cells (cytotoxic and regulatory), myeloid-derived suppressor cells (MDSC), natural killer cells (NK cell), neutrophils and other stromal components (including the extracellular matrix and cancer-associated fibroblasts (CAF)) are shown.

In recent years it has become increasingly evident that studying the tumor microenvironment (TME), in addition to studying cancer cell transformation, is crucial to understanding tumor growth, progression and dissemination. TME is a complex and heterogeneous milieu where cancer cells and stromal cells (including immune cells and other cells resident in the tissue) interact with each other and with the extracellular matrix (ECM), Figure 1. One of the critical elements of the TME is the tumor’s interaction with the host immune system. Hanahan and Weinberg described evasion of the immune system as one of the hallmarks of cancer [1]. The importance of the stromal microenvironment in tumor progression was also recognized in the classical paper by Paget [2]. It has become clear that the tumor stromal component, and specifically, the host immune system, contributes to tumor growth, and new therapeutics are now being aimed at altering the immune system as a cancer target (see reviews [3,4]).

The cellular components of the TME can vary in different regions of the tumor [5], as well as between patients and even between tumors in a single patient [6]. Each of these cellular components has its own behavior in terms of migration, proliferation, differentiation, apoptosis, adhesion, and response to treatment. Cancer is so difficult to treat partly because of this degree of complexity that results in a highly unpredictable tumor behavior, partially due to the complex microenvironment [7], emphasizing the pressing need for personalized treatment for individual patients. Mathematical and computational modeling techniques provide a powerful tool in understanding the TME and predicting cancer progression [8]. Below, we provide a brief overview of the role of the immune system in cancer and introduce computational approaches to study the tumor immune microenvironment (TIME).

## 2. Immune System Biology and Cancer

The immune system consists of two major parts: the innate immune system, and the adaptive immune system. The innate immune system is the body’s immediate defense against foreign antigens. The immunity via the innate immune system is nonspecific and short-lived, whereas the adaptive immune response is a late-stage immune response that is highly specific and can provide long-lasting defense [9]. The innate immune cells have pattern recognition receptors that recognize entities that are non-self and then induce an inflammatory response. The innate immune response is immediate, although some studies suggest these cells also have the capability for memory [10], and may be followed by an adaptive immune response [11]. The adaptive immune response is more specific than the innate immune response and can be antibody-mediated or cell-mediated, with T- and B-cells as the key cell types driving this response [12]. T-cells are a type of lymphocyte that matures in the thymus, with several different subtypes that play a distinct function in immune response [13]. Cytotoxic T-cells kill cancer cells [13], T-helper cells assist other cell types during the immune response, and T-regulatory cells (Treg) play an important role in immunological tolerance. In the case of cancer, in addition to normal antigens (self), cancer cells express antigens unique to the tumor, which can result in an immune response. The computational models discussed in this review will largely focus on the adaptive immune system.

Although the immune system is well equipped to eliminate abnormal cells, cancer cells have several ways to evade the immune system. For example, cancer cells can become invisible to the immune system by downregulating major histocompatibility complex (MHC) class-I receptors on their cell surface, and in turn, not presenting the mutation associated antigen for detection by T-cell receptors. Cancer cells attract regulatory T-cells (Treg) [14] and myeloid-derived suppressor cells (MDSC) [15] to the tumors; an abundance of these cell types results in an immunosuppressive environment [16]. Furthermore, receptors such as CTLA-4 on Treg can bind to CD80 and CD86 on T-cells and antigen presenting cells (APC), which inhibits co-stimulation of these cells, and results in T-cell suppression. Treg can further inhibit the adaptive immune response by interfering with B-cell function and releasing immunosuppressive cytokines such as IL-10. In fact, under certain conditions, the immune response can contribute to tumor growth instead of inhibiting it [17]. Another way that tumors escape detection is through chronic inflammation [18]. During chronic inflammation, T-cells eventually lose their effectiveness over the course of the infection, called T-cell exhaustion [19]. T-cell exhaustion is also frequently found in the tumor microenvironment through the PD-L1/PD-1 pathway [20]. PD-1 blockage enhances T-cell and NK (Natural Killer) cell activity in tumors [21,22]. Several immune checkpoint inhibitors are currently being used in treatments of patients with cancer [23,24].

Innate immune cells, such as macrophages, neutrophils, and eosinophils, have also been shown to decrease or enhance tumor growth depending on their polarization state. Macrophages have a great deal of plasticity but are usually classified in one of two types: an M1-type that is immuno-enhancing, and an M2-type which is immuno-suppressing; though there is a spectrum of states between M1 and M2 [25]. Studies have shown that high numbers of tumor-associated macrophages (TAM) can lead to a worse clinical outcome [26]. TAM have been shown to promote tumor growth by increasing vascularization, cancer cell migration, cancer cell survival, and immuno-suppression [27]. Macrophages are recruited to hypoxic areas of the tumor [28], and aid in tumor progression [26,29]. Cancer cells recruit macrophages through Colony Stimulating Factor 1 (CSF1), and high CSF1 concentrations are correlated with poor prognoses [30]. Macrophages can be converted to TAM within the tumor by secreted factors, such as c-c chemokine receptor type-2 (CCR2) [31], which causes them to exhibit an M2-like phenotype [32]; this conversion of macrophages leads to a distinct subpopulation [33]. TAM secretion of c-c chemokine ligand type-18 (CCL18) can enable the epithelial-to-mesenchymal transition of breast cancer cells [34,35]. These TAM have also been shown to be associated with invasion, extravasation and metastasis [36–38]. Thus, M2-type macrophages are currently being targeted with therapeutics for tumor treatment [39]. Neutrophils are less abundant in tumors, but they are becoming more recognized for their duel role in the immune response to cancer [40]. Eosinophils are also commonly found within tumors [41], and have been found to enhance T-cell infiltration [42]. These cells also have a duel role in the immune response, and can promote or suppress tumor growth [43]. There is a complex interaction between cancer cells and the immune system. Thus, it is important to understand the conditions in which tumors are eliminated or enhanced by the immune system.

As is evident from the complex mechanisms of immune response and immune evasion described above, modeling the immune system is a challenging task [44]. For the specific case of cancer, immune cells can be found within the TME, the lymphatic system and the lymph nodes, resulting in spatial complexity. Molecular and cellular components themselves are complex and have patient specific features such as unique lymphocyte antigen receptors. In addition, different functions of the immune system occur at different time scales, ranging from minutes to years. For example, intracellular signaling occurs in minutes, whereas memory cells exist on the order of years. Revealing this complexity across different scales as discussed above is very challenging or impossible to achieve in an experimental setting. Thus, computational modeling platforms provide a powerful tool to complement experimental measurements for better understanding of the immune system in cancer. In the following section, we provide an overview of the current computational modeling approaches for the study of cancer.

## 3. Overview of Computational Modeling Methodologies including Agent-Based Modeling

**Figure 2.**
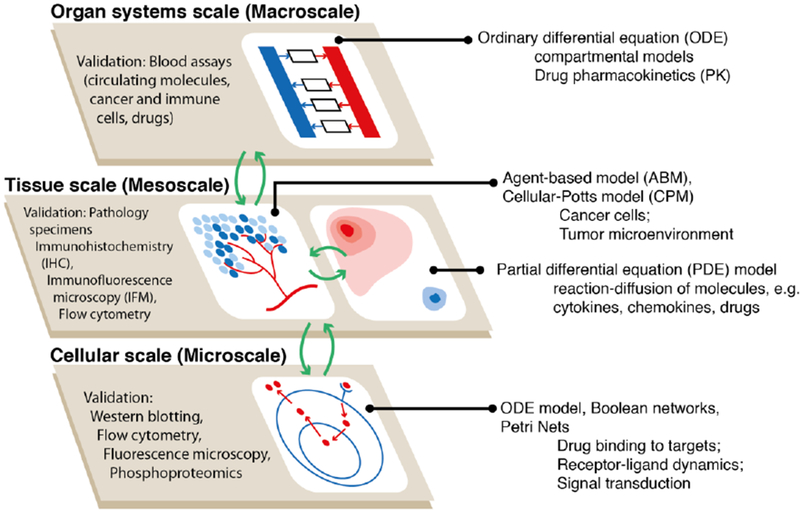
Using hybrid models to study immuno-oncology. While agent-based models are ideal tools to recapitulate the spatio-temporal dynamics of cancer cells and the tumor microenvironment at the tissue scale, the mechanisms at other biological scales can be efficiently embodied using other types of mathematical representations; however, agent-based models (ABM) can also be used at any scale. Such multi-scale hybrid models increase the flexibility in model construction, improve computational performance, and enhance model credibility by allowing comparison between model output and a wide range of experimental and clinical observations

Computational modeling has provided great insight into studying intra-tumor heterogeneity [45] and the interplay between the tumor and the microenvironment [46]. Modeling has the benefit of providing a quantitative time- and cost-effective means to study the physical and chemical interactions in tumor initiation and growth. Modeling efforts complement experimental platforms by providing an understanding of clonal dynamics and microenvironmental cues over time. There are several ways to classify mathematical/computational models in general, and cancer models in particular. One is deterministic vs. stochastic; another is continuum vs. discrete models. Deterministic models have an end state that does not change as long as the initial conditions remain the same, whereas stochastic models have randomness included, resulting in differences in end states, even with the same initial conditions. Continuum models treat cells as concentrations of cell types, whereas discrete models (such as agent-based or particle models) consider discrete cells; the cell behaviors, including interactions between cells, can be described as deterministic or stochastic. A multiscale setting (called a hybrid model, illustrated in Figure 2) can include both approaches, i.e., the discrete modeling of cells and continuous modeling of molecular species, such as oxygen, growth factors, chemokines, microRNAs, and drugs, but appropriate linking and calibration of such hybrid models should be performed. For continuum-based models, temporal ordinary differential equations (ODE) and spatio-temporal partial differential equations (PDE) have been used to model the immune response in cancer, e.g., [47–53]; here, we focus on discrete agent-based models (ABM) and hybrid models.

From another standpoint, the models that describe the immune system can be broadly categorized into top-down and bottom-up, and previous reviews have focused on computational modeling of the immune system [44,54]. The top-down approach models populations of cells, not single entities, and uses the mean behavior at the macroscopic level. ODE and PDE models are examples of this type of modeling where individual interactions are not simulated. Stochastic differential equation models are also a part of this class. On the other hand, the bottom-up approach focuses on the microscopic level. The model tracks each agent (e.g., a cell) and its interactions with the surrounding environment, and emergent behavior arises from all the entities and their local behavior. Features such as stochastic behavior, spatial distribution, and heterogeneity of entities are inherent to bottom up models, and thus, easier to capture with this approach. Drawbacks of these models are that they require more computational power because they track individual agents and their interactions over time and space; also, there are computational limitations on the number of agents that can be considered; it is thus impossible to consider an entire organ or patient. Therefore, both approaches will need to be combined to achieve both spatial cellular and sub-cellular resolutions and whole patient pharmacokinetics and pharmacodynamics.

Agent-based models are an example of a bottom-up approach with applications in immunology and immune related diseases such as cancer [55]. An agent-based model is a discrete mathematical and computational framework that is capable of capturing emergent behavior of its interacting agents, defined as large-scale spatio-temperal patterns resulting from local spatial interactions between agents. The behavior and function of these agents are driven by the information they sense in their local environment and the rules of the agent-based model. Some of the characteristics that separate agent-based models apart from other rule-based modeling systems (in which outcomes are based on a set of rules that govern decisions) are that (1) they are spatial, (2) they incorporate agents that interact with other agents and their environment, (3) they may incorporate stochasticity, (4) they are modular, and (5) they produce emergent behavior [56]. These models allow the individual agents to adapt to their local environment (i.e., agents are adaptive instead of reactive), and take part in local interactions with other agents [57]. These result in complex aggregate behavior stemming from simple rules and emergent properties from agent interactions. Agent-based models can be lattice-based or lattice-free, depending on whether agents reside and move on a (regular or irregular) spatially discretized lattice, or have their locations and velocities represented by continuous variables, usually governed by forces in the environment. For example, in lattice-based ABM, agents are placed on a lattice structure that defines the locations of cells and their neighbors for cellular interactions. There are several model types that, although they are not explicitly characterized as agent-based, are reviewed here for completeness; those include cellular automata, Potts models, and Petri net models.

Agent-based models are particularly suitable for capturing spatially-varying events and heterogeneities [58], and for understanding the immune system’s function. With this aim in mind, several investigators have developed agent-based models of diseases with involvement of the immune system. Several ABM have simulated the immune system’s involvement in maintaining homeostasis and disease conditions, such as bacterial infections [59], fungal infections [60], abnormal systemic inflammatory response [61], ulceration [62], allergens [63], ischemia [64], tuberculosis [65], sepsis [66], and wound healing [67]. For cancers, such models include tumor growth and invasion [68], as well as specific cancer types such as hepatocellular carcinoma [69], breast cancer [70], melanoma [71], colorectal [72], lung cancer [73,74], and metastasis [75]. Software packages have been developed based on the ABM framework to study the immune system; these include ImmSim [76–79], Immunogrid [80,81], Simmune [82], Cycell [83], and PhysiCell [84].

Now we discuss the latest agent-based and hybrid models that investigate the effects of the immune system on cancer progression and immunotherapy, see Table 1. In order to be included in the review, the work needs to have an immune component, a tumor component, and include ABM. We limited our focus to papers that were published within the last ten years. However, we also included some studies on diseases other than cancer where we feel that the methodology is relevant and could be applied to cancer; we also refer to a few general software tools that can be readily adapted to cancer.

We break the review into the following sections, although there may be significant overlap:

(1) Models focusing on immune-related tumor mechanobiology
(2) Models focusing on tumor-associated vasculature in the immune response
(3) Models focusing on tumor-associated lymphatics and lymph nodes
(4) Models focusing on tumor immunotherapy
(5) Models focusing on tumor-enhancing immune cells
(6) Models focusing on intra-tumor heterogeneity

### 3.1. Models Focusing on Tumor Mechanobiology

Changes in the tumor extracellular matrix (ECM) have been known to contribute to tumor progression and metastasis [85], with several computational models focusing on investigating glioma invasion [86–88], but less is known about its contribution to immune response. Computational modeling has been used to shed light on the interactions between the ECM and the immune system in cancer dynamics. A hybrid agent-based model was used to investigate the role of cellular adhesion to the ECM in tumor and immune system dynamics [89]. Frascoli et al. found that the greater the motility of the cancer cells, the more likely they will escape from immunotherapy. They also found that intermediate levels of adhesion in general led to less successful outcomes, but these results were variable.

Kather et al. used ABM to investigate the combination of adoptive cell transfer and therapy that permeabilized the fibrotic stromal component in colorectal cancer [72]. Adoptive cell transfer is a therapeutic strategy that aims to increase the number of immune cells to strengthen immuno-surveillance and counter tumor development. Kather et al. simulated various conditions of immune surveillance. In their model, T-cell killing of tumor cells occurred in a purely stochastic manner, with killing probability representing the effect of tumor specificity, immunogenicity, stimulatory and inhibitory effects all in one parameter. An immune rich environment promoted immune escape, but tumor growth slowed in a lymphocyte deprived environment. Tumor control was observed in a subgroup of tumors with less stroma and a high numbers of immune cells. They found that high levels of fibrosis and low numbers of lymphocytes reduced overall survival. Their findings were validated with data from colorectal cancer patients, where low density stroma and high lymphocyte level correlated with better overall survival. In this study, Kather et al. simulated the effect of immunotherapy by boosting the number of immune cells by 2–8 fold. Therapy was intended to enhance fibrotic stromal permeability; this was implemented by modifying the corresponding parameter by a factor of 4% to 16%. The model predicted that optimal tumor eradication requires a combination of therapeutics aiming at both activating adaptive immune system and stromal depletion.

### 3.2. Models Focusing on Tumor-Associated Vasculature in the Immune Response

Tumor-associated vasculature is an important aspect of the tumor-immune complex because it not only provides oxygen and nutrients for the tumor to grow, but it is also the source of tumor dissemination via circulating tumor cells (CTC), and recruitment for many immune cells, such as monocytes/macrophages and T-cells. Studies have aimed to provide a better understanding of these processes [90,91]. An ABM of Early Metastasis (ABMEM) framework was used to model the interactions between tumor cells, platelets, neutrophils, and endothelial cells [92]. Receptor binding to Mac-1 (macrophage antigen-1) by endothelial cells, platelets, or tumor cells leads to reactive oxygen species (ROS) production by neutrophils. Uppal et al. examined two types of platelet inhibition: inhibition of thromboxane, inhibition of adenosine diphosphate (ADP) receptors and inhibition of both [92]. They found that thromboxane inhibition alone resulted in the best outcome.

Alfonso et al. developed an agent-based model of immune cell-epithelial cell interactions in breast lobular epithelium [93]. The model investigated the effect of menstrual cycle length and hormone status on inflammatory response to cell turnover in breast tissue. Blood vessels were homogeneously distributed in the intra- and interlobular stroma. The model accounts for myoepithelial and luminal cells. Cellular processes (i.e., epithelial cell proliferation, cell death via effector cells, programmed cell death, removal of dead cells, immune cell motility, and inhibition of effector cells by regulatory cells) are modeled as stochastic events. Effector CD8+ cells are the only cells responsible for killing of damaged epithelial cells. Regulatory CD4+ and CD8+ cells act by inducing inactivation of effector dependent response. Chemokines from damaged epithelial cells activate the immune cells. Immune cells become ineffective when such chemokines are absent, or due to the suppression via regulatory cells. The outcome of the model identified novel prognostic information for breast cancer, such as the number of immune clusters being associated with the degree of epithelial damage.

### 3.3. Models Focusing on Tumor-Associated Lymphatics and Lymph Nodes

Reddy developed the first mathematical model of the lymphatic system in 1977 [94]. In recent years, various computational modeling approaches have been used to study the lymphatic vessels [95–99] and lymph nodes [100–102] in general, and in the application to infectious disease [103,104], and simulating fluid and chemokine transport in the lymphatic system as it relates to health and disease conditions [105]. Agent-based models have more recently been used to simulate various processes that occur during an adaptive immune response in a lymph node. Meyer-Hermann developed an ABM of germinal centers of the lymph node [106,107]. The authors studied B-cell germinal center reactions and how they contribute to germinal center deregulation [106]. They expanded this model to study B-cell affinity maturation in the lymph node germinal centers [108]. They found that competition for T-cell rescue and increased refractory time leads to a more robust affinity maturation.

A series of studies on modeling of T-cell behavior in the lymph node have been conducted by Bogle and Dunbar [44,109–112]. They modeled T-cell trafficking, activation, and proliferation in the lymph node paracortex using an agent-based approach. The model included chemokine and cytokine gradients. Using this lattice-based approach, they were able to model the movement and behavior of T-cells in the lymph node paracortex [112]. In the next step, they expanded the agent-based model of the lymph node paracortex in three dimensions to include T-cells and dendritic cells (DC). The model allows simulation of a large number of T-cells at physiologic densities. The virtual lymph node can shrink or swell, depending on the dynamics of cell trafficking. The model was able to simulate T-cell activation in agreement with in-vivo observations, and provide new understanding on T-cell-DC interactions. Not all the parameters of the model were experimentally measured; thus, the model can be refined by more accurate measurement of those parameters [110]. Next, the authors built on their previous models to simulate T-cell ingress and egress, as well as chemotaxis in the lymph node, by incorporating new numerical methods. The new model allows simulation of expansion and contraction of T-cells in the lymph node paracortex during an immune response. The ability to model chemotaxis could be useful in studying other biological processes involving chemotaxis [111].

Moreau et al. constructed a virtual lymph node using agent-based modeling to study T-cell activation by synapses (long-lasting contacts) and kinapses (transient interactions) [113]. The model incorporated T-cell migration and T-cell-DC interactions. Additionally, virtual fluorescence-activated cell sorting (FACs) profiles were obtained from modeling by visualizing T-cell proliferation. This virtual lymph node model provides new opportunities for understanding the mechanisms of T-cell regulation in infection or vaccine application [113].

The ABM developed for studies of the lymphatic system thus far mainly focus on the lymph node, and in particular, T-cell processes. Folcik et al. developed the basic immune simulator (BIS), which is an agent-based platform that includes parenchymal tissue, secondary lymphoid tissue and the lymphatic/humoral circulation [114]. Using agent-based and hybrid models, lymph node dynamics are studied in the context of infectious diseases and cancer. Kim and Lee used a hybrid model to study the efficacy of preventative cancer vaccines. The model comprised two compartments for interactions of tumor and immune cells at the tissue site and in the draining lymph nodes [115]. Jacob et al. developed a three-compartment ABM that includes lymph nodes, blood vessels, and organ/tissue. The model was used to study immune response against viruses in these compartments [116]. Marino et al. developed a hybrid model where the lymph node and blood compartment were simulated using ordinary differential equations and the lung compartment was simulated using agents. They focused on the formation of granulomas in the lung, which are organized structures of immune cells in the lung, and are a hallmark of infection. The model focused on the recruitment of APCs in the lymph node from the lung for *Mycobacterium tuberculosis* (Mtb, the causative bacterium of TB) infection [117]. In another study, they investigated the role of DC in Mtb infection [118]. The growth and dissemination of bacteria were highly affected by CD8+ and CD4+ T-cell proliferation rates and DC migration. Such multiscale models allow the study of tissue level dynamics during adaptive immune response [118], and although they focus on infectious disease, many of the components and processes involved in anti-cancer immunity and adaptive immunity against infection are shared. For example, T-cells specific to tumor antigens are primed and expanded in a similar fashion to that in which T-cells specific to foreign antigens are during their response to infection; the immune suppressive mechanisms that cancer cells hijack to evade immune surveillance are also deployed during an immune response against infection to prevent excessive tissue damage. Since the body reacts similarly in response to an infection as it does in response to cancer (e.g., activation of similar signaling pathways), cancer models can heavily borrow from this literature.

### 3.4. Models Focusing on Tumor Immunotherapy

A variety of cancer immunotherapy strategies exist that range from boosting the overall immune response to specifically targeting cancer immunity. Some examples of immunotherapies are treatment vaccines, adoptive cell transfer, and immune checkpoint inhibitor treatments. Agent-based and hybrid models are developed to help understand these therapies when applied separately or in combination with other cancer treatments. One type of therapy that has been explored is cancer vaccines. Therapeutic cancer vaccines treat existing cancers by delivering immunogenic and tumor specific antigens to the patient to induce cellular and/or humoral anti-tumor immunity. Pennisi et al. have developed several hybrid models investigating the immune system effects on tumors. They developed a hybrid model to study the development of lung metastases from mammary carcinoma [75]. Pennisi et al. also developed a hybrid model MetastaSim to simulate the protection against lung metastases in mouse using Triplex cell vaccine [73]. In this simulation, macrophages could capture tumor-associated antigen and immunocomplexes, breaking them down and eliminating them from the system. This vaccine elicited a combination of three stimuli: the p185neu antigen expressed by the HER2/neu gene, allogeneic major histocompatibility complex (MHC) molecules, and IL-12 which enhances antigen presentation. Using this model, after calibration and validation, the authors were able to evaluate different protocols of vaccine administration. The simulation results suggested that in order to maximize protection while reducing the number of administrations, the vaccination strategy should include a significant dosage early on and a few recalls afterwards.

Dreau et al. developed an ABM model of solid tumor progression to understand the interplay between solid tumor growth, tumor vascular growth, and the host’s immune system [119]. The model includes tumor and immune cells, vasculature, tumor cell proliferation, and immune system response. Their model supported immunotherapy as an effective cancer treatment in individuals with functioning immune systems. They concluded that a strong immune response limits tumor growth in a way that cannot be achieved under a weaker immune response. Another study focused on the role of T-cells in the effectiveness of response to immunotherapy in B-16 melanoma [120]. The model includes macrophages, DC, tumor vasculature, and interactions between these components. It was found that early entry of T-cells effectively eliminated the tumor and was dependent on CD137 (a co-stimulatory protein that helps in tumor rejection [121]) expression in tumor vasculature.

Oncolytic virus therapy is a strategy that utilizes viral infection to kill cancer cells, but not normal cells, with the potential of enhancing T-cell recruitment to the tumor and increasing their access to cancer cells. Several computational models have examined the conditions of success for this type of therapeutic in silico [122]. Walker et al. developed an agent-based model of pancreatic tumors to study the synergy between chimeric antigen receptor (CAR) T-cell therapy and oncolytic virus therapy [123]. CAR T-cell therapy is one type of adoptive cell transfer treatment involving genetically engineered T-cells specifically targeting cancer cells, and has been the subject of several computational models [124]. The agent-based model recapitulates treatment mechanisms including cancer specific CAR T-cell recruitment to the tumor site via vasculature and the injection and spread of oncolytic virus. Rohrs et al. demonstrated the ability of the model to track the dynamics of cancer cells and stromal cells in space in the presence of the treatment combinations; optimization of the combination therapy requires more accurate calibration [124].

Immune checkpoint inhibitors are used in cancer immunotherapy that enhances anti-tumor immune response by targeting cancer immune evasion mechanisms. In many cancer types, tumor neoantigens are sufficiently immunogenic to promote the expansion of antitumor immune cells [125]; however, these immune cells are not functional due to the inhibitory signals from molecules adaptively induced during cancer development [24,126]. Among them, one of the most prominent mechanisms is PD-1/PD-L1 interaction, where T-cells are suppressed through PD-1 signaling upon contact with induced PD-L1 in the tumor microenvironment. Gong et al. developed an ABM of tumor-immune interaction in 3D to study the spatio-temporal dynamics of cancer cells and cytotoxic T-cells [127]. In this study, the inhibitor to the checkpoint molecules were modeled as a factor which modulates the parameter governing the suppression of tumor specific T-cells by PD-L1+ cancer cells. They found that patient responsiveness to such therapy could be associated with the level of mutational burden of the cancer and antigen strength among patients. They also found that tumor growth is insensitive to the vascular density of the tumor core. From these results, a scoring method was proposed to predict anti-PDL1 treatment efficacy in patients.

### 3.5. Models Focusing on Tumor-Enhancing Immune Cells

While the immune system has evolved to kill off tumor cells, there are many ways in which cancer cells can avoid immune detection. In addition, there is mounting evidence that immune cells can stimulate tumor growth under certain conditions. Several agent-based models have focused on understanding the tumor-enhancing contributions of the immune system. Enderling and colleagues explored the interactions between tumor cell death and the immune system using a cellular automata model focused on the interplay between cancer stem cells and the immune system [128]. They showed that immune system-induced tumor cell death led to stem cell selection, and thus, more aggressive tumors [128,129]. In this model, even though immune cells effectively killed off tumor cells, they also affected progenitor cells. This resulted in the creation of a space for cancer stem cells to proliferate and produce more cancer stem cells. This ultimately resulted in a larger stem cell population and a more aggressive tumor.

Several studies have specifically focused on the tumor-enhancing contribution of immune cells, such as tumor-associated macrophages (TAM). Macrophages are one of the most abundant immune cells found in tumors, but their population is heterogeneous [130]. M1-type macrophages have been shown to be tumor inhibiting, whereas M2-type macrophages have been shown to be tumor enhancing [26]. One model looked at the transition from the M1 to M2 macrophage phenotype on tumor growth and then predicted targeted therapies [131]. Knútsdóttir et al. used a hybrid model to investigate epidermal growth factor (EGF) and macrophage colony-stimulating factor 1 (CSF-1) signaling between macrophages and cancer cells during macrophage aggregation [132]. They found that CSF-1/CSFR1 autocrine signaling affects the ratio of tumor cells to macrophages during tumor growth. In a further study, they found that the macrophage/tumor cell ratio was most sensitive to the strength of EGF signaling, but usually maintained a 1:3 ratio [133].

Another ABM of triple-negative breast cancer examined the tumor enhancing effects of macrophages [134]. Norton et al. investigated the interplay between tumor growth, blood vessel recruitment and macrophage recruitment through tumor vasculature. They observed that while macrophages increase tumor growth, excessive macrophage recruitment conversely leads to a decrease in tumor growth due to the inhibition of proliferation resulting from overcrowding.

### 3.6. Models Focusing on Intra-Tumor Heterogeneity

Intra-tumor heterogeneity and the characteristics of the tumor microenvironment are found to have important implications in the outcome of disease progression [135]. Patients often have varied responses to treatment because each patient is unique in their genome, microbiome, disease history, lifestyle, and environment. The case of tumors is especially complex, because this heterogeneity is observed not only between tumors, but also between subpopulations of cells from the same tumor, resulting in different response to drugs [136]. While capturing this degree of heterogeneity may be difficult in experiments and clinical trials, especially the temporal dynamics of spatial heterogeneity, computational models are especially suited to tackling this challenge. This section focuses on the models that have aimed to capture intra-tumor heterogeneity.

A 2D agent-based model was used to study the interactions between an avascular tumor and immune cells (NK cells and cytotoxic T-cells) [137]. They examined the effects of cancer cell proliferation on overall tumor growth under two conditions: the first, where cancer cells do not consider the microenvironment when deciding when to proliferate, and the second, where they proliferate based on the number of healthy cells surrounding them. Tumor-immune cell interaction can have three outcomes: tumor cell death, immune cell death, or no cell death based on the state of the tumor. The predicted growth of the tumor was then compared to a xenograft tumor growth. Spatial heterogeneity was also examined in a different model where cancer cells use glycolysis instead of oxidative phosphorylation to increase their energy production. In order to study how this increased energy production affects the surrounding stroma, a combination of computational modeling and in vitro/in vivo experiments was used [138]. They used agent-based modeling to understand tumor growth in a vascularized area of the tumor. They found that tumors develop spatial patterns where macrophages and tumor cells coexisted in areas with high levels of oxygen, but that only tumor cells survived in ischemic regions. They then used an in vitro tissue-mimetic system to create the directional gradients for oxygen and lactate, which also allowed for the co-culture of tumor cells and macrophages.

Figueredo et al. created a series of hybrid models to study the interplay between the immune system (including macrophages) and tumor cells [139,140]. An agent-based on-lattice model for tumors was created using Chaste (Cancer, Heart and Soft Tissue Environment), part of the Virtual Physiological Human (VPH) Toolkit; the model consists of three layers: a diffusible layer, a cellular layer, and a subcellular layer [141]. The diffusible layer consists of diffusible species such as oxygen, the cellular layer consists of normal cells, tumor cells, and macrophages, and the subcellular layer governs apoptosis and cell-cycle in each cell. In this model, macrophages were M1-like, they supported the immune system, and aided immunotherapies. They investigated the growth of the tumor under oxygen-dependent proliferation. They found that the emergent behavior of agent-based models allowed for the generation of additional tumor architectures over other modeling methodologies [142].

## 4. Discussion and Emerging Applications

The immune system is made up of many interacting components that together drive a complex spatio-temporal behavior during immune response. Thus, agent-based modeling is particularly suitable for understanding the immune systems function in health and in disease conditions such as cancer. Here, we reviewed the latest agent-based and hybrid models that investigate the contributions of the immune system to cancer growth and the effect of immunotherapy. In this context, we focused on models of immune-related tumor mechanobiology, tumor-associated vasculature, tumor-associated lymphatics, tumor immunotherapies, tumor-enhancing immune cells, and finally, models focusing on intra-tumor heterogeneity. Overall, ABM can generate novel hypotheses to be validated and refined by future experiments. Development and refinement of multiscale agent-based models along with experiments through an iterative process can improve our understanding of biological processes in cancer and lead to the identification of novel prognostic and predictive biomarkers that can improve therapies and help design and interpret the results of clinical trials [143].

Models investigating the tumor-enhancing effects of the immune system can provide useful insights into managing tumor-immune interactions. Since the tumor microenvironment can be very heterogeneous, care must be taken to appropriately model cell-cell interactions between cancer, stromal, and immune cells, the extracellular matrix, and the secreted factors. Accurate data from in vitro and in vivo experiments must be used to understand the transition from tumor-inhibiting to tumor-enhancing immune cell types. In addition, since immune cells such as macrophages and T-cells are usually recruited to the tumor by secreted factors, an evolving tumor vasculature is necessary to accurately model these processes. Agent-based models of the tumor enhancing effects of the immune system can help us better understand how to prevent or revert the immune system back into a tumor-inhibiting phenotype. Thus, these models will help improve immunotherapies for cancer treatment.

**Figure 3.**
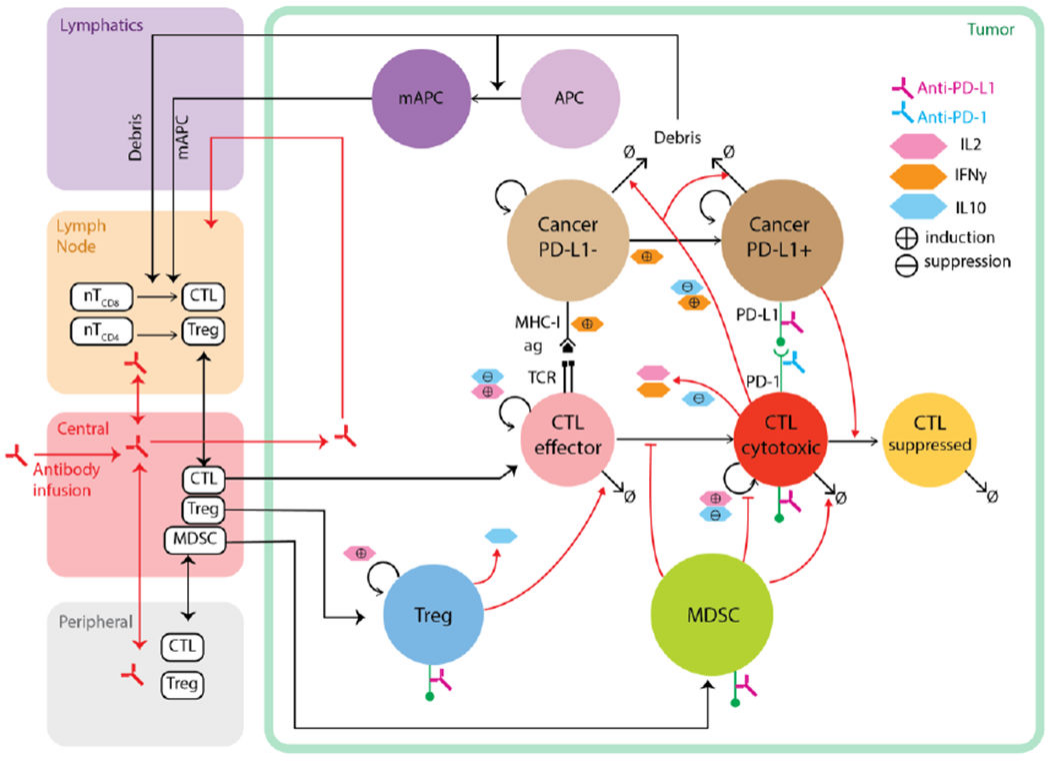
Diagram of a multi-compartment hybrid model capturing tumor development and anti-tumor immune response. Dynamics of cells and pharmacokinetics of drug (e.g., antibody) in the lymphatics, tumor-draining lymph node, central (blood) and peripheral compartments are modeled using ordinary differential equation systems. Spatial dynamics of cells and molecules in the tumor compartment are captured using agent-based model and partial differential equations. Death of cancer cells produces antigens which drive maturation of APC and their migration to the tumor draining LN, where CD8+ and CD4+ T-cells go through priming and proliferation before they enter blood circulation and extravasate to the tumor microenvironment. Effector CD8+ T-cells can be further activated and expanded when they encounter tumor antigens. These cytotoxic cells kill cancer cells and also release various cytokines, including IL2 which drives further proliferation of T-cells, and IFNγ which is proinflammatory and induces PD-L1 expression on cancer cells. PD-L1 can then bind to PD-1 molecules on cytotoxic T-cells, resulting in T-cell exhaustion. Both PD-L1 and PD-1 molecules are potential targets for immune checkpoint blockade antibodies. Regulatory cell types in the ABM include Treg and MDSC, which can inhibit cytotoxic T lymphocytes (CTL) through different mechanisms.

One limitation of the immunotherapy studies mentioned above is that although the models are quantitative in many aspects, the modules governing drug delivery and response is relatively qualitative or semi-quantitative in nature. This could potentially be resolved by combining spatio-temporal agent-based models with traditional model types, such as Physiologically Based Pharmacokinetic (PBPK) models to track drug distribution in different physiological compartments, and pharmacodynamic (PD) models for individual cellular agents to represent effects of drugs on target cells [144]. Such hybrid quantitative systems pharmacology (QSP) models can be utilized not only as a platform for basic science research, but also as a potential complement to the clinical research and drug discovery pipeline. A schematic of such a hybrid model based on the research in our own group is presented in Figure 3. Compared with continuous models, the discretely represented agents allow the flexibility to track intra-tumor heterogeneity, such as tumor neoantigen profile, T-cell clonality and local expression of immune checkpoint molecules with preferred levels of granularity. By running multiple simulations in parallel using different parameter values and initial conditions accounting for genetic background and environmental exposure, the models can represent cohorts of patients with desired population scale heterogeneity. These properties render agent-based and hybrid models a powerful platform for conducting virtual clinical trials.

Computer models provide large-scale predictive power by allowing us to simulate clinical trials with sufficient details to study response to various conditions. Using these models, it is possible to test and predict drug failures in simulations rather than in patients, which could result in improved drug design, reduced risks and side effects, and can dramatically decrease costs of drug development. Importantly, models can predict how the immune-tumor system evolves during the course of the treatment [136]. The challenge is that available data for individual patients is limited. To address this problem, machine learning approaches can be used to build statistical models based on available patient data, and these models can be employed to simulate virtual populations to predict the effects of therapies [145]. These approaches have already been expanded to identify biomarkers and find important mutations that affect response to treatment with drugs in cancer cell lines [146–148].

Mechanistic models are another suitable approach that provides large-scale predictive capabilities based on the available information on the interactions between various components of a biological system. These models can be discrete or continuous. A certain type of model is chosen based on the application and availability of the data [149,150]. Depending on the type of the model, predictions can be made for the behavior of the signaling system in a qualitative, semi-quantitative or quantitative manner. For example, for quantitative predictions of signaling and regulatory gene networks, continuous variables need to be modeled on continuous time scales using ODEs [151].

Such detailed modeling requires knowledge of important biological reactions at every step. This can only be achieved in several iterative steps that include the implementation of various components such as signaling events and defining values for related parameters and appropriate initial conditions. In recent years, numerous models have been developed that simulate individual signaling pathways [152–154]. The challenge is that these models often do not fully capture crosstalk mechanisms that are crucial in predicting patient response to treatment, as each drug perturbs multiple biological processes. ODE-based models can be combined with agent-based models to capture the dynamics of the system being modeled in a more complex fashion. Stochasticity is one of the main advantages of agent-based models, as it applies to biological processes [155]. In comparison to ODE models that make predictions of concentrations and other events over time, ABM allows the study of each agent, as it interacts with other agents in their proximity and the ways in which that affects the large-scale behavior. These models, however, are computationally more expensive. There are also challenges in validating results from ABM due to insufficient spatio-temporal data on tumor development [156].

One aspect that is typically missing from mechanistic knowledge-based models including QSP and ABM is an input from high-throughput data, genomic or proteomic; such data can inform the models and can supplement the data obtained at the cellular and tissue levels [157]. Examples include immune landscape information from different sources including patients’ databases such as TCGA (The Cancer Genome Atlas) [33,158,159]. Another source of “Big Data” for parameterization and validation of the models, including ABM, will be the emerging methodologies of digital pathology, such as using multiplex immunofluorescence (mIF) of patients’ biopsies and resected tumors, with subsequent analysis of cellular and molecular spatial patterns. Steps in this direction are already underway [160]. Another source of data is image-based, using microCT, confocal, multiphoton, and super resolution microscopy, both ex vivo and in vivo; examples include imaging entire tumor vasculature with subsequent computer simulation of blood flow and molecular transport [161,162].

In addition to modeling approaches used to simulate response to treatment, virtual patients are a key component of virtual clinical trials. A virtual population has the characteristics of the original patient population but also includes individual diversity, usually comprising parameter sets weighted by a clinical or response distribution [163]. This diversity allows testing a broad range of responses that can be missed in a clinical trial. In contrast to traditional clinical trials that can only be performed after costly and lengthy development, in silico trials could be performed at every stage of the drug development. In silico design of treatments can be conducted with data-driven or mechanistic-based (knowledge-based) approaches [164]. It should be noted that in silico clinical trials require integration of data at different scales via a multi-model approach using virtual patients. Similar to a traditional clinical trial, rigorous statistical approaches are needed at various steps of virtual clinical trials. The ability to test treatment via in silico clinical trials can significantly reduce the cost and increase the efficacy of drug development.

The main challenge in the way of predictive models for virtual clinical trials is the availability of input data for the model for each patient. Detailed knowledge about the situation at the start of the simulation can significantly affect the predictive power of that model. Such input information is being generated at a growing rate and a lower cost. Furthermore, proteomic data enable the modeling of interactions of different subgroups of cells from the same tumor with each other as well as immune cells and other stromal cells, allowing modeling tumors for individual patients. Computational models can make predictions for the optimal treatments, making it safer, faster, and cheaper to complement current clinical trials. These models will improve by continuous comparison of predicted and actual response to therapy. Additionally, as more detailed information on biological parameters and disease mechanisms become available, the accuracy of the models will increase.

Currently, only 1 in 10s of clinical trials results in drugs that make it to the market [165]. The process takes 10–12 years, costing billions of dollars, sometimes with low effectiveness when used by real patients [136]. Although virtual clinical trials with virtual patients and virtual cohorts cannot replace clinical trials, they can inform design of such trials to improve success rates and increase the efficacy of the process of drug development. Virtual trials can also better address the need for personalized therapy [166]. Finally, combining agent-based models and data-driven artificial intelligence (AI) methods (e.g., machine learning, including deep learning), we can better understand the gap between preclinical findings and clinical outcomes.

In summary, in silico modeling and specifically agent-based modeling are powerful tools of cancer systems biology and cancer immune systems biology. Combined with novel measurement methodologies and increasing amounts and sophistication of data available from clinical trials, they should bring a better mechanistic understanding and predictive capabilities of therapeutic interventions in cancer, including immunotherapies. The field is ripe for conducting predictive virtual clinical trials as a prerequisite to clinical trials in patients.

## Figures and Tables

**Table 1.** Summary of Section 3: ABM and hybrid models discussed in each section.

3.1. Models Focusing on Immune-Related Tumor Mechanobiology		3.2 Models Focusing on Tumor-Associated Vasculature in the Immune Response		3.3 Models Focusing on Tumor-Associated Lymphatics		3.4 Models Focusing on Tumor Immunotherapy		3.5 Models Focusing on Tumor-Enhancing Immune Cells		3.6 Models Focusing on Intra-Tumor Heterogeneity	
Study	Ref	Study	Ref	Study	Ref	Study	Ref	Study	Ref	Study	Ref
Cellular adhesion to ECM	(Frascoli et al. 2016) [89]	Early metastasis	(Uppal et al. 2017) [92]	Germinal centers of LN	(Meyer-Hermann et al. 2002, 2005) [106–108]	Lung met in mammary carcinoma	(Pennisi et al. 2009) [73]	Cancer stem cell-immune cell interaction	(Hillen 2013) (Enderling 2012) [128,129]	Tumor, NK cell, cytotoxic T-cell interactions	(Pourhasanzade et al. 2017) [137]
Adoptive cell transfer in colorectal cancer	(Kather et al. 2017) [72]	Immune-epithelial cell interactions in breast epithelium	(Alfonso et al. 2016) [93]	T-cell behavior in LN	(Bogle et al. 2010, 2012, 2008) [109–113]	Effect of vaccine on lung metastasis	(Pennisi et al. 2010)[75]	Effect of M1 and M2 macrophages on tumor growth	(Wells 2015) [131]	Effect of stroma on tumor spatial patterns	(Carmona-Fontaine et al. 2013) [138]
				T-cell activation in virtual LN	(Moreau, 2016) [113]	Immunotherapy in solid tumors	(Dréau et al. 2009) [119]	Signaling between macrophages and cancer cells	(Knútsdóttir et al. 2014, 2016) [132,133]	Immune cell, macrophage, tumor cell interactions	(Figueredo 2011, 2013) [139,140]
				Model of LN to study cancer vaccines	(Kim et al. 2009) [100]	Role of T-cells in response to immunotherapy	(Pappalardo et al. 2011) [120]	Effect of macrophages on TNBC tumor growth	(Norton et al. 2018) [134]	Tumors under oxygen-dependent proliferation	(Figueredo 2013, 2014) [141,142]
				Immune response against viruses	(Jacob et al. 2011) [116]	Effect of different therapies on pancreatic tumors	(Walker et al. 2016) [123]				
				Recruitment of APCs in the LN from lung	(Marino et al. 2011) [117]	Spatio-temporal dynamics of tumor-immune cell interactions	(Gong et al. 2017) [127]				
				T-cell trafficking and proliferation	(Marino et al. 2016) [118]						
